# Manufacture and Characterization of Heat-Resistant and Heat-Insulating New Composites Based on Resol Resin–Carbon Fibers–Perlite for the Built Heritage Protection

**DOI:** 10.1155/2019/8791010

**Published:** 2019-02-21

**Authors:** George Soupionis, Loukas Zoumpoulakis

**Affiliations:** Department of Chemical Engineering, National Technical University of Athens, Athens 9 Heroon Polytechniou Str., Zografou Campus, 157 73, Greece

## Abstract

Composite materials were created for usage as reinforcement and to protect the building envelope based on today's global conditions such as climate change. Composite materials were manufactured using phenol-formaldehyde resin (case of resol) as a matrix, carbon fiber as reinforcement (7.5%*v*/*v*), and perlite (10%*w*/*w*) as a low thermal conductivity component, to combine high mechanical properties with good heat resistance and good thermal insulation properties. The structure of these new materials was examined through scanning electron microscopy (SEM) and elemental analysis (SEM-EDS). The addition of perlite (10%*w*/*w*) in the resite matrix (without fibers) increased the flexural and shear strength of the composite materials. On the other hand, the composite materials with fiber reinforcement show that the perlite reduces the flexural and shear strength due to the additional interfaces which were created. During heat treatment at 473 K, carbon fibers had the smallest weight loss followed by perlite while the resite matrix (i.e., the cured resol) shows the greatest weight loss. It is noted that the role of perlite is to stabilize the mass of the resite matrix during heat treatment. The composite material with carbon fibers and perlite is a heat-resistant material with only 2% weight loss at 473 K for 1 hour and shows a low coefficient of thermal conductivity, making it a new material in the direction of heat-insulating materials.

## 1. Introduction

Considering it holistically, built heritage constitutes a living organism of global importance since it carries both the history of humanity and a landmark function for the future. At the same time, today's global conditions such as climate change that disrupt past experience-based performance expectations of materials in a specific yet now rapidly changing localities, resulting, for example, in abrupt material degradation [1], brings to the fore the need for new compatible materials, new methods, and reconstruction techniques in protecting the heritage [2, 3].

The insulation and the reinforcement of the building envelope in a way that is compatible with traditional construction are set as top priorities [4], particularly by bearing in mind that they are responsible for the energy loss and the stability of the structural system of historic buildings. Of particular attention is the key role of the structural elements. These may include external and internal load-bearing brick or masonry walls, mud walls, or timber-framed walls; columns of stone, cast iron, or concrete; stone, brick, or concrete vaults; and timber, iron or steel beams, trusses, or girders. In modern cultural heritage (built after 1940), the most common structural elements are cast iron and concrete—something that intermediates between the old and the new materials and techniques in terms of compatibility. On the other hand, as far as older buildings are concerned, one is faced with inherent difficulties in finding appropriate and compatible solutions including materials with good mechanical properties, heat resistance, and thermal insulation [5]. It is in such a complex restoration and preservation milieu that composite materials find their new and augmented role.

Composite materials typically consist of a thermosetting polymer matrix with glass fibers as reinforcement. In this form, they are applied in many sectors [6–8] such as shipbuilding, automobiles, and construction. They have an advantage compared to other materials due to their low density. However, for applications where high strength and rigidity are needed, glass fibers are not the reinforcement that can be used. For applications with high requirements, where a high modulus of elasticity is required [9, 10], for instance, in the aerospace and automotive industries, in high mechanical requirements, and sporting goods, carbon fibers or aramid fibers are used since both fiber categories, in addition to high tensile strength, also have a high modulus of elasticity.

After the cross-linking of the macromolecules, the thermosetting resins—the cured—are in a solid state, and they are insoluble in solvents. The most common resins in this category are phenolic resins, epoxy resins, and polyester. Phenolic resins are produced by polycondensation reactions of phenol-formaldehyde through gradual polymerization with acid catalysts as is the case with novolac resins or base catalysts as is the case with resol resins. Modern technology exhibits increasing demands for composite materials with excellent mechanical properties; however, other properties are often required as well, such as thermal.

Materials that are used for thermal insulation usually have thermal conductivity coefficient (*λ*) less than 0.1 W/mK [11]. The thermal conductivity of a thermal-insulating material depends on the volume of enclosed air in the pores of the material, the size and structure of the pores, the moisture content, and so on. Thermal-insulating materials are divided into two categories: inorganic and organic. Organic heat-insulating materials are polymeric materials, such as polyurethane, extruded and/or expanded polystyrene [11], and phenolic resins (Bakelite), mainly in a foam form [11]. Phenol-formaldehyde resins exhibit excellent mechanical properties and also thermal-insulating properties and heat resistance, in contrast to epoxy resins. A remarkable inorganic thermal-insulating material is perlite, which is a low-density material and, as an additive, contributes effectively in the reduction of the weight of the composite material [7]. It also contributes to thermal-insulating and heat-resistant properties and improves the mechanical properties of the polymeric matrix. In addition, as an inorganic material, in case of fire, it contributes to the limitation of released gases, to the prevention of flame propagation and to the containment of the blaze [8, 9, 12].

The aim of the paper is to present materials that can help in the stability of the buildings and possess good mechanical properties as well as heat resistance properties which can safeguard the structural system of the buildings against high temperatures by providing adequate thermal insulation by protecting the building envelope. To achieve this purpose, composite materials were manufactured with polymeric matrix, which has high mechanical properties combined with good thermal resistance and satisfactory thermal insulation properties. To this objective, heat-resistant components are selected for the composite material, in particular, (a) phenol-formaldehyde resin (resol) as a matrix, (b) carbon fibers as a reinforcing agent, and (c) perlite as a component with a low thermal conductivity coefficient. Composite materials of different compositions and proportions of their components are manufactured, and pictures of the samples are taken through scanning electron microscopy (SEM) including elemental analysis (EDS). Furthermore, the mechanical properties of the specimens are measured in terms of flexural strength and shear resistance, while their heat resistance and their thermal conductivity coefficient are determined.

Τhe features that constitute the composite material help to protect the building envelope. Thermal resistance together with thermal conductivity will protect the building in extreme conditions such as extreme weather events. The typical building materials that are used disrupted in the presence of water or even moisture, and the consistency of materials is lost. By manufacturing a heat-resistant material and with good thermal conductivity, the building is protected by the presence of such phenomena. Also, mechanical properties keep the building safe from any displacements due to contraction and expansion of materials or even to some small seismic vibrations.

### 1.1. Experiment

The resol resin used as a matrix to construct the composite material is laboratory-synthesized through phenol polycondensation (p.a., MERCK) with formaldehyde (p.a., Riedel-de Haen, solution 36.5%) and barium hydroxide (Fluka) as a catalyst. The carbon fibers used had a monofilament number of 3000 tex and a density of 1740 kg/m^3^. In addition, the perlite is a mineral silicate (70-75% SiO_2_, 12-15% Al_2_O_3_, 3-4% Na_2_O, 3-5% K_2_O, and below 1% for Fe_2_O_3_, MgO, and CaO) [13]. In this case, commercial perlite with a density of 120 kg/m^3^ was used after being ground to a particle size of <3 · 10^−4^ m and then dried at 443 K for 2 hours. For the manufacture of the composite material, the carbon fibers which have been mentioned above were preimpregnated in a 1 : 6.6 wt% solution of phenol-methanol (Fluka) in a specially configured fiber wrapping system. After preimpregnation, the impregnated carbon fibers were heated at 343 K for 1 hour to remove the methanol, and the resin was partially cured at 343 K for 1 hour after which the preimpregnated carbon fibers were cut into laminates (prepregs). To manufacture the composite specimens, the casting technique was employed as follows. For better dispersement of perlite, a resol-methanol solution (70 : 30 wt) was added, and the mixture was placed in an ultrasonic bath for 2 hours. The carbon fibers were placed in an open mold, and the resol with the dispersed perlite was poured over. After the mold was filled, it was placed in a temperature-controlled furnace so when the resol was cured, it would be transformed into resite. The heating program was 10 hours at 343 K and 2 hours at 353 K, and then the temperature increased every hour for 283 K until it reaches 403 K with a residence time of 1 hour at each temperature. Finally, the specimen was left for 2 hours at 423 K to complete the cure, and then the postcuring process was given an hour at 443 K. To prevent the curling of the specimens during curing, when 363 K was reached, a plate was placed at the top open side of the mold. However, in order to determine the coefficient of thermal conductivity, larger specimens are required. The manufacture of these composite materials was done in a 0.28 m diameter circular mold using 0.003 m chopped carbon fibers, 1700 kg/m^3^ (R&G Faserverbundwerkstoffe GmbH), following the previous procedure.

It is noted that the density of the respective materials is required to calculate the ratio of components of the composite material (by weight (*w*/*w*) and by volume (*v*/*v*)). The density of the resite and the density of the composite specimens were determined by weighing and measuring their dimensions.

### 1.2. Scanning Electron Microscopy (SEM)

The scanning electron microscope used is the FEI QUANTA 200 model. Initially, all specimens were gold plated and then placed on a special sampler, which was inserted into the instrument at a suitable irradiation site under high vacuum and at a voltage of 15 kV. The analysis of the microstructure of the specimens was carried out in magnifications ranging from 100x to 5000x, while in selected samples, an energy distribution analysis (EDS) was performed for their qualitative and quantitative elemental analyses.

### 1.3. Mechanical Properties

The mechanical properties of the composite materials, specifically bending strength and shear strength, were determined by the three-point method according to ASTM D790-71 and D2344-65T (BS EN ISO 14125: 1998). For the bending and shearing tests, the specimen is placed horizontally on a support span, and the load is applied to the center by the loading nose, a special dynamometer, which, by applying pressure, measures the resulting deflection in proportional indication. The result corresponds to a force in a table given by the instrument manufacturer, and then the bending strength *σ*_*b*_ (MPa) and the shear strength *τ*_*b*_ (MPa) are calculated:

(1) $$\begin{array}{c} \sigma_{b}=\frac{3P_{\max}l_{s}}{2{bd}^{2}},\\ \tau_{b}=0.75\frac{P_{\max}}{bd}, \end{array}$$

where *P*_max_ is the maximum load, as the specimen breaks (N); *l_s_* is test length (m); *b* is width (m); and *d* is thickness (m).

### 1.4. Heat Resistance

The heat resistance of each material was determined by weight loss after heating in a furnace. The heat resistance is determined separately in the individual components of the composite material as well as in the composite material. The furnace had been heated to 473 K and not higher since phenolic resins decompose and begin to lose their properties above this temperature. The weighed materials were placed therein in the presence of atmospheric air. The materials were weighed every 1 hour for 5 hours, and the weight loss of the materials was determined.

### 1.5. Determination of the Thermal Conductivity Coefficient

**Figure 1:**
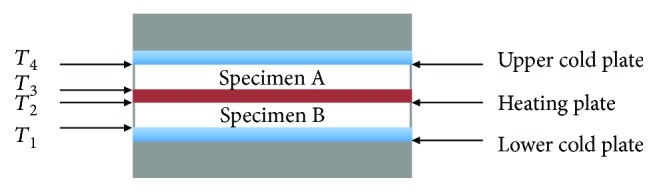
The operating principle of the device for measuring the thermal conductivity coefficient *λ*. *T*_1_, *T*_2_, *T*_3_, and *T*_4_ are temperatures measured by the corresponding thermocouples. The heated metal plate indicates the heating position of the built-in electrical resistance through an external regulator.

The coefficient of thermal conductivity of the composite material with 10%*w*/*w* perlite and 7.5%*v*/*v* carbon fibers is determined in an appropriate composition. The two specimens of the composite material were cast into large disks in a diameter of 28 cm, which is equal to the diameter of the metal plates of the sampler. The measuring device is based on the method of DIN 52 612 and includes a suitable heating regulator as well as an appropriate sampler. The latter consists of three identical (0.28 m) metal plates where the central plate is the thermal plate (with a suitable built-in resistance) and provides heating through an external heating regulator while the other two are the cold plates and lie above and below the central one. The two identical test pieces of the composite materials are placed between the cold metal plates so as to measure the temperature. The complete layout of the sampler is well insulated at the top and bottom, as well as circumferentially. The sampler configuration and the temperature measurement locations are shown in Figure 1. Measurements are made isothermally at 317 K, and the following equations are applied:

(2) $$\begin{array}{c} \lambda =\frac{\Phi S_{m}}{2A\left(\Theta_{\text{wm}}-\Theta_{\text{cm}}\right)}, \end{array}$$

(3) $$\begin{array}{c} \Phi =P\frac{x}{y}, \end{array}$$

where *A* is the sample surface (m^2^); *S*_*m*_ is sample thickness (m); Θ_wm_ is average temperature of hot surfaces of specimen A and B where Θ_wm_ = 0.5 (*T*_3_ + *T*_2_) (m); Θ_cm_ is average temperature of cold surfaces where Θ_cm_ = 0.5 (*T*_1_ + *T*_4_); Φ is capacity resistance of the heating plate calculated by equation (3) (coefficient 2 because we have 2 samples); *x* is the time the system emits heat over a certain period of time; *y* is the time period that the system emitted heat for time *x*; and *P* is the power outside the thermal heater (W).

## 2. Results and Discussion

To manufacture the composite specimen resite resin, one direction carbon fibers and perlite were used as shown in Table 1.

The densities of the above materials were as follows: resite *ρ*_*p*_ = 1190 kg/m^3^, R90-P10 composite *ρ*_*Σ*1_ = 1040 kg/m^3^ (expected density from the rule of the mixtures with the proportions of the individual components per weight *ρ*_*Σ*1M_ = 1080 kg/m^3^), and of the composite material R90-CF7.5-P10 *ρ*_*Σ*2_ = 1060 kg/m^3^ (expected density *ρ*_*Σ*2M_ = 1130 kg/m^3^). Therefore, replacing an amount of the resite matrix with perlite (10% by weight) leads to a lower density of the composite material (R90-P10), because the perlite density is about 10 times lower than that of the resite.

**Figure 2:**
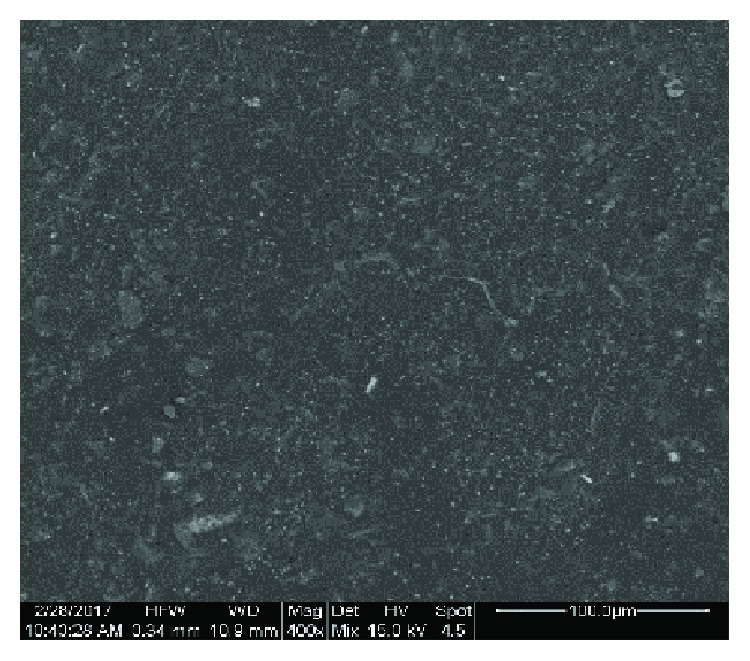
SEM image of specimen with carbon fibers and perlite (R-P10-CF7.5) at magnification 400x.

**Figure 3:**
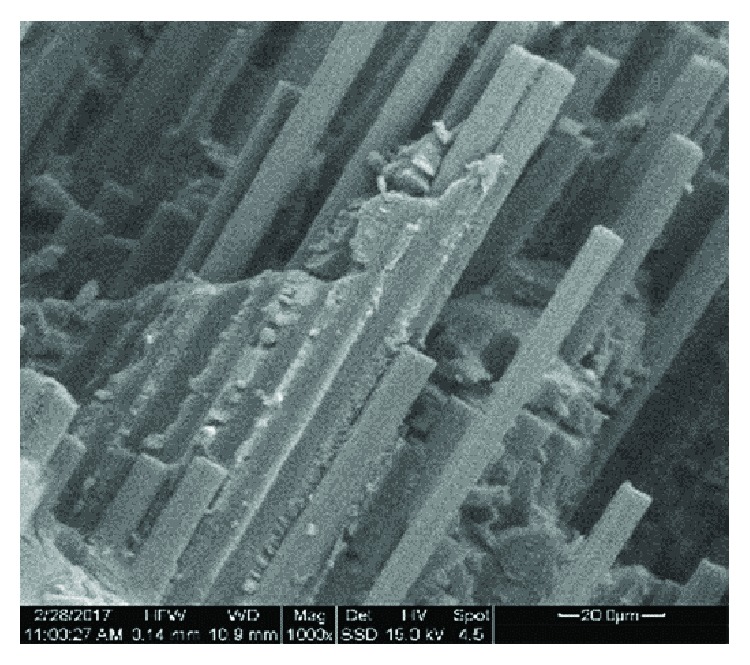
SEM image focused on the breaking surface of the specimen with carbon fibers and perlite (R-P10-CF7.5) at magnification 1000x.

**Figure 4:**
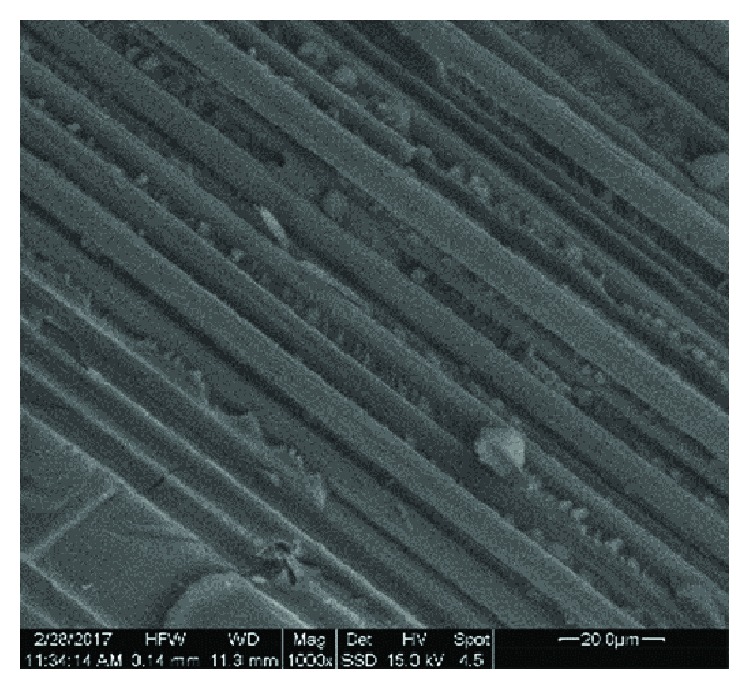
SEM image of carbon fibers (R-CF7.5) focused on the breaking surface where the carbon fibers are distinguishable.

Figure 2 shows the SEM photo of a resite specimen with carbon fiber and perlite (R-P10-CF7.5) at a magnification of 400x, Figure 3 is at a magnification of 1000x focused on the ruptured surface where the carbon fibers are visible, and the Figure 4 specimen of resite with carbon fiber (R-CF7.5) at 1000x magnification is focused on the rupture surface where the carbon fibers are distinguishable. From Figures 2 and 3, it is noted that the dispersion of the perlite is homogeneous. Also, from Figures 3 and 4 which are focused on a region where the carbon fibers are visible, the parallel arrangement of the fibers in the composite specimen and the homogeneous coating of the fibers from the matrix resite can be observed.

Table 2 shows the results of elemental analysis SEM-EDS of the composite materials. The analysis focused at the rupture area of the composite material (Figure 3), in order to determine the role of the perlite and its dispersion percentage due to the additional interfaces that were created. It is also clear that the structure of the raw materials remains unaffected with regard to each other. Figure 2, which focuses on the surface of the composite material, shows silicon and aluminum which are the main components of perlite whereas in Figure 3, which shows the rupture surface of the composite material, the carbon fibers dominate and the presence of silicon is limited (small Si and no Al).

**Figure 5:**
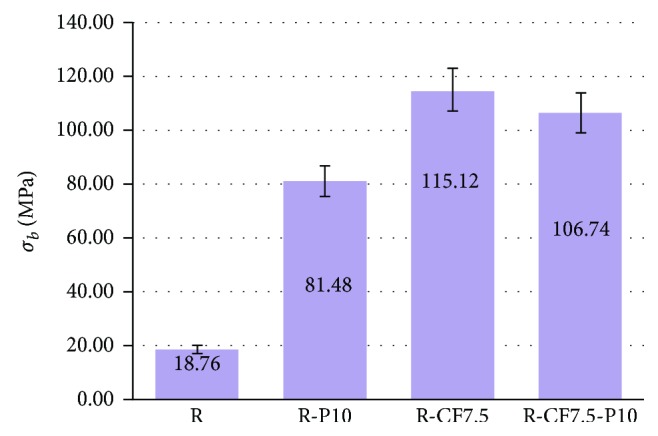
Bending strength of resite (R) and composite materials of resite-perlite (R-P10), resite-carbon fibers (R-CF7.5), and resite-perlite-carbon fibers (R-P10-CF7.5). Variation of values ± 7%.

**Figure 6:**
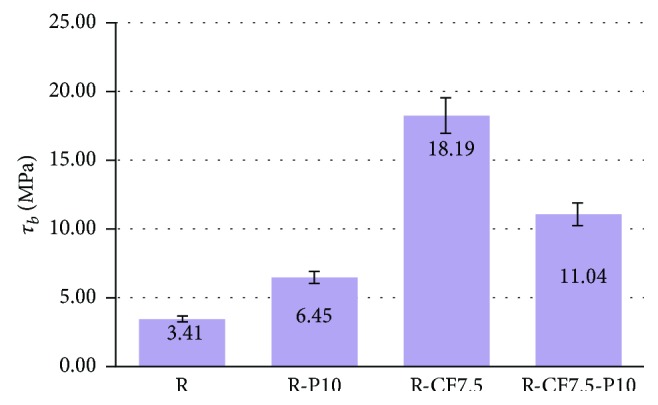
Shear strength of resite (R) and composite materials of resite-perlite (R-P10), resite-carbon fibers (R-CF7.5), and resite-perlite-carbon fibers (R-P10-CF7.5). Variation of values ± 7%.

Figures 5 and 6 show the bending strength and shear strength of the following materials: resite (R), resite with perlite (R-P10), resite with carbon fibers (R-CF7.5), and resite with carbon fibers and perlite (R-P10-CF7.5). From Figure 3, it is evident that due to the addition of perlite 10%*w*/*w* in the resite matrix (R-P10), the bending strength increases by 434% (approx. 5 times).

In the case of composite materials containing carbon fibers, the perlite (R-P10-CF7.5) reduces the flexural strength by 7% relative to that of composites without perlite (R-CF7.5). Accordingly, Figure 4 shows that shear strength is increased by 52% (approximately 2 times) for the specimen containing perlite 10%*w*/*w* (R-P10) relative to the resite (R) specimen. In the case of composite materials with carbon fibers and perlite (R-P10-CF7.5), the shear strength is reduced by 38%. Consequently, perlite constitutes a material that enhances the matrix, while the fibers enhance the composite to a greater extent than the granular material. Simultaneous involvement of the perlite in fiber-reinforced composite leads to additional interfaces which contribute to the reduction of the mechanical properties of the material. The mechanical properties show that this material can be used for restraint of the structural system of a building as reinforcement.

**Figure 7:**
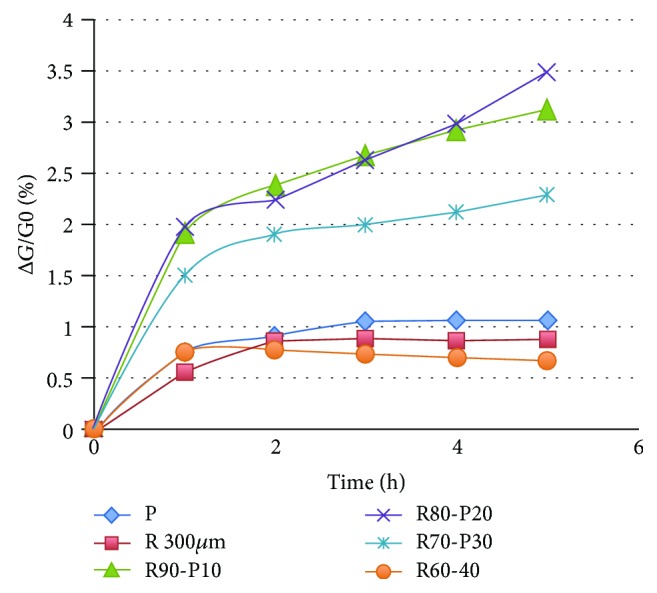
Thermal behavior at 473 K for 5 h of perlite and resite-perlite samples at various percentages of perlite.

**Figure 8:**
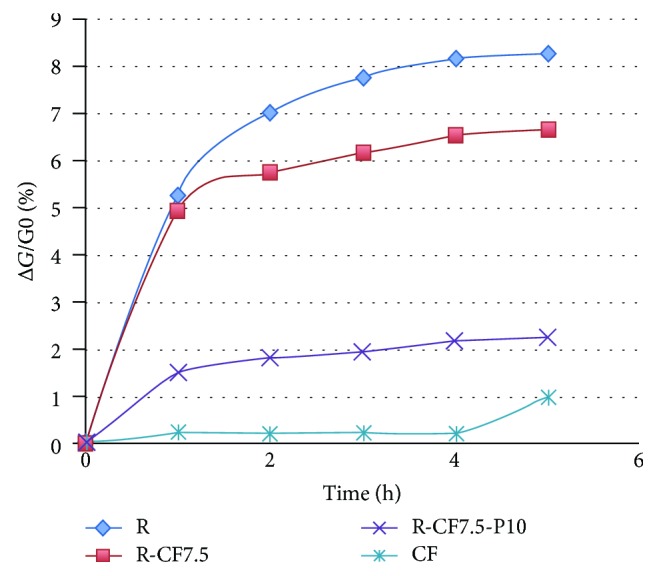
Thermal behavior at 473 K for 5 h of materials: resite, carbon fiber, and composite material R-P10-CF7.5.

Figures 7 and 8 display the percentage of weight losses of various materials relative to the corresponding matrix material of the composite materials for their heat treatment at 473 K versus time. As shown in Figure 5, commercial perlite, as well as perlite with a particle size < 3 · 10^−4^ m, shows a minimum weight loss (~1%) up to 1 hour, possibly due to moisture content. The composite material resite-perlite in a proportion of 60-40 wt has a similar behavior to commercial perlite. By reducing the percentage of perlite and at the same time increasing the rate of resol, weight loss is slightly increased to 3.5%. Given that the weight loss of the resite matrix, based on Figure 6, is 8.3% for 5-hour heating at 473 K, the expected weight loss of the matrix of the composite materials of Figure 5 is calculated: 4.9% (R60-P40), 5.8% (R70-P30), 6.6% (R80-P20), and 7.4% (R90-P10). The significantly lower weight loss values based on Figure 7 relative to the previous expected values indicate the stabilizing role that perlite performs for the retention of mass of the resite matrix during the heat treatment.

**Figure 9:**
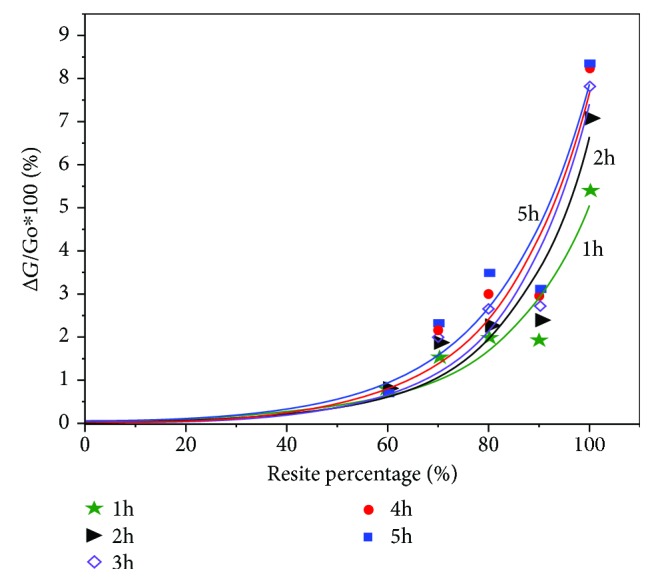
Weight loss of the composite material of peroxide with additional perlite at various rates, at 200° C, as a function of the content of the resite.

Based on Figure 8, the least weight loss occurs in carbon fibers, while the largest is found in the resite matrix. Concerning the composite materials that contain carbon fibers, those that contain perlite clearly show a lower weight loss, which confirms the stabilizing role of perlite, which means it safeguards the structural system of a building against high temperatures.  Figure 9 shows the weight loss of the composite material with additional perlite at various percentages, as a function of the resite content for heat treatment at 473 K for up to 5 hours. It has been found that for low percentage wt of resite (60%*w*/*w*), the time parameter (*t* = 1 − 5 h) practically does not affect the weight loss of the material. By increasing the percentage of the resite, the time parameter progressively affects the weight loss of the material, and the effect of time is maximized for 100%wt of resite.

The experimental points were adapted to the exponential function: *y* = *A*^∗^exp(*R*_0_^∗^*x*) with *R*^2^ = 0.9.

Table 3 presents the results of the coefficient of thermal conductivity, *λ*, for composite material of phenolic resin– (resite–) carbon fibers–perlite and the values of *λ* for various thermoset polymers as building materials in general and more specifically as heat-insulating materials [15].

The value of the thermal conductivity coefficient of the composite material (R-CF7.5-P10) is 4.5 to 1.5 times lower than the thermosetting polymeric materials, while it is 4.5 to 3.2 times higher than the typical foamed heat-insulating polymeric materials. Therefore, the specific composite material is located approximately in the middle between the two categories of materials on the basis of the value of *λ*. The values of the coefficient of thermal conductivity (*λ*, W/mK) of the individual components of the composite material are the following [17]: phenolic resin (phenolic cast resins) *λ* = 0.15, carbon of simulated graphite structure *λ* = 1.7, carbon fibers PAN (Cytec Thonel, T300) *λ* = 8, and perlite *λ* = 0.031. Applying the rule of mixtures (*w*/*w*), the *λ* value of the composite material (R-CF7.5-P10) takes the value *λ* = 1.69 (assuming *λ*_*ίνες*_ = 1.7) and *λ* = 7.4 (assuming *λ*_*ίνες*_ = 8). These values (*λ* > 1) are within the lower limit of the range of thermal conductive materials. By comparing these values, as expected by the rule of mixtures, the experimentally determined value of the composite material (*λ* = 0.16) shows a significant deviation. In particular, the much lower experimental value of *λ* shows that the composite material, in terms of thermal conductivity, behaves as a new material with respect to its individual components and even by 10.6 or 46.2 times lower in the direction of the heat-insulating materials.

Nowadays, one must apply two individual processes with independent and separately procured materials in order to secure the thermal protection and the reinforcement of a building envelope, respectively, adding in this way some additional load to the building. Here, a new material is shown, namely, one which can concurrently have both properties, i.e., both high strength and resistance to thermal loads, and thus, it can serve both purposes at the same time.

## 3. Conclusions

- The replacement of a quantity in the resite matrix with perlite (10%*w*/*w*) leads to a lower density of the composite material (R90-P10) since the density of perlite is about 10 times lower than that of resite
- The SEM images and the SEM-EDS elemental analysis of the specimen R-P10-CF7.5 show the presence of perlite on the surface, while at the rupture surface of the composite material, carbon fibers dominate, and the presence of silicon is limited
- The addition of 10%wt of perlite (R-P10) increases the strength of the material in bending and shearing. In the case of composite material that is fiber reinforced, it is found that the perlite involvement reduces the bending and shear strength due to the additional interfaces created in the composite material mass. Perlite is enhancing the matrix, while the fibers enhance much more than the granular material (perlite)
- Regarding the heat treatment of materials at 473 K, carbon fibers have the smallest weight loss, while perlite suffers less weight loss, closely followed by the composite, with a proportion of 60-40 wt, with a value of 1% for 5 hours. The resite matrix exhibits the greatest weight loss of all materials at 8.3% for 5 hours. In fiber-reinforced composite materials, the smallest weight loss can be found in the one containing perlite, which confirms the stabilizing role of the substance. The graph of the weight loss of the composite material with perlite (no carbon fibers), as a function of the content of the resite, follows an exponential function, where for a low percentage %wt (60%*w*/*w*), the time parameter (*t* = 1 − 5 h) does not practically affect the weight loss of the material. Composite material (R-CF7.5-P10) is a heat-resistant material (weight loss 2% for 5 hours at 473 K)
- From the coefficient of thermal conductivity of the composite material (R-CF7.5-P10), it was found that, on the one hand, it is located approximately at the middle between the two categories of materials (thermoset and typically foamed heat-insulating polymeric materials). Base on the *λ* value (thermal conductivity coefficient) the material behaves as a new material compared to its individual components and even lowers in the direction of heat-insulating materials
- For these composites, modifying the proportions of their components and the technique to create a foam matrix, the thermal conductivity coefficient is expected to be further reduced as a typical heat-insulating material
- Such materials can help in the stability of the modern cultural buildings due to their mechanical properties; and their heat resistance properties safeguard the structural system of the buildings against high temperatures providing adequate thermal insulation. As such, composite materials can play a vital role in keeping the legacy alive both as a reminder of the past as well as a live and constitutive part of our modern world

## Figures and Tables

**Table 1:** Compounds of resol resin–carbon fiber–perlite.

Composite material	Resol^∗^ (%*w*/*w*) (R)	Perlite (%*w*/*w*) (P)	Carbon fiber (%*v*/*v*) (CF)
R	100	0	0
R-CF7.5	100	0	7.5
R90-CF7.5-P10	90	10	7.5
R90-P10	90	10	0
R80-P20	80	20	0
R70-P30	70	30	0
R60-P40	60	40	0

^∗^ or resite, after the resol curing.

**Table 2:** Elemental analysis of SEM-EDS for specimens of resite with carbon fibers and perlite (R-P10-CF7.5) and resite with carbon fibers (R-CF7.5).

Material	Figure	C		O		Na		Al		Si		Au	
		%wt	%at	%wt	%at	%wt	%at	%wt	%at	%wt	%at	%wt	%at
R-P10-CF7.5	2	69.60	79.33	21.29	18.21	0.38	0.22	0.55	0.28	3.33	1.62	4.87	0.34
R-P10-CF7.5	3	73.18	84.28	17.00	14.70	—	—	—	—	0.79	0.39	9.02	0.63
R-CF7.5	4	78.25	85.00	18.10	14.76	—	—	—	—	—	—	3.65	0.24

**Table 3:** Coefficient of thermal conductivity of composite material of phenolic resin– (resite–) carbon fibers–perlite in comparison with other organic thermal-insulating materials.

Material	Polymeric matrix	Additive	Carbon fibers (%*w*/*w*) (CF)	Coefficient of thermal conductivity *λ* (W/mK)	
	Resol (R)^∗1^ Polyurethane (PU) (%*w*/*w*)	Perlite (%*w*/*w*) (P)			
R-CF7.5-P10	90	10	10 (*ή* 7.5%v/v)		0.16
Cured phenolic resin	100	0	0	Typ 31^∗2^ Τyp 13.5^∗2^	0.31 [14] 0.72 [14]
PU	100	0	0	Solid Foam	0.25 [15] 0.05 [15]
Extruded polystyrene	100	0	0	DOW	0.035 [16]^∗3^

^∗1^ or resite, after resol curing. ^∗2^ formation groups of phenol-formaldehyde resins with additives. Group I: types for general application (type 31: wood-flour) and group IV: types with increased electrical properties (type 13.5: with mica, i.e., phyllosilicate mineral) [17]. ^∗3^ foamed extruded polystyrene as a material with uniform small and closed cells.

## Data Availability

Data are available on request. Alternatively, authors may make data available on request through a data access committee, institutional review board, or the authors themselves. In this case, they should name who should be contacted to request the data (e.g., the ethics or data access committee) and provide appropriate contact details. The corresponding author's email address is gsoupionis@central.ntua.gr.
